# Remodeling of the human skeletal muscle proteome found after long-term endurance training but not after strength training

**DOI:** 10.1016/j.isci.2023.108638

**Published:** 2023-12-05

**Authors:** Eric B. Emanuelsson, Muhammad Arif, Stefan M. Reitzner, Sean Perez, Maléne E. Lindholm, Adil Mardinoglu, Carsten Daub, Carl Johan Sundberg, Mark A. Chapman

**Affiliations:** 1 Department of Physiology and Pharmacology, Karolinska Institutet, 171 77 Stockholm, Sweden; 2 Science for Life Laboratory, KTH – Royal Institute of Technology, 171 77 Stockholm, Sweden; 3 Department of Women’s and Children’s Health, Karolinska Institutet, 171 77 Stockholm, Sweden; 4 Department of Biology, Pomona College, Claremont, CA 91711, USA; 5 Division of Cardiovascular Medicine, Department of Medicine, Stanford University School of Medicine, Stanford, CA 94305, USA; 6 Centre for Host–Microbiome Interactions, Faculty of Dentistry, Oral & Craniofacial Sciences, King’s College London, London WC2R 2LS, UK; 7 Department of Biosciences and Nutrition, Karolinska Institutet, 171 77 Stockholm, Sweden; 8 Science for Life Laboratory, 171 65 Solna, Sweden; 9 Department of Laboratory Medicine, Karolinska Institutet, 141 52 Huddinge, Sweden; 10 Department of Learning, Informatics, Management and Ethics, Karolinska Institutet, 171 77 Stockholm, Sweden; 11 Department of Integrated Engineering, University of San Diego, San Diego, CA 92110, USA

**Keywords:** Biological sciences, Health sciences, Medicine, Omics

## Abstract

Exercise training has tremendous systemic tissue-specific health benefits, but the molecular adaptations to long-term exercise training are not completely understood. We investigated the skeletal muscle proteome of highly endurance-trained, strength-trained, and untrained individuals and performed exercise- and sex-specific analyses. Of the 6,000+ proteins identified, >650 were differentially expressed in endurance-trained individuals compared with controls. Strikingly, 92% of the shared proteins with higher expression in both the male and female endurance groups were known mitochondrial. In contrast to the findings in endurance-trained individuals, minimal differences were found in strength-trained individuals and between females and males. Lastly, a co-expression network and comparative literature analysis revealed key proteins and pathways related to the health benefits of exercise, which were primarily related to differences in mitochondrial proteins. This network is available as an interactive database resource where investigators can correlate clinical data with global gene and protein expression data for hypothesis generation.

## Highlights

• Endurance-trained individuals display a vastly altered skeletal muscle proteome
• Minimal muscle proteome differences between strength-trained and untrained humans
• Few sex differences in the skeletal muscle proteome identified
• 8% of skeletal muscle gene-protein pairs were significantly correlated

## Graphical abstract

**Figure:**
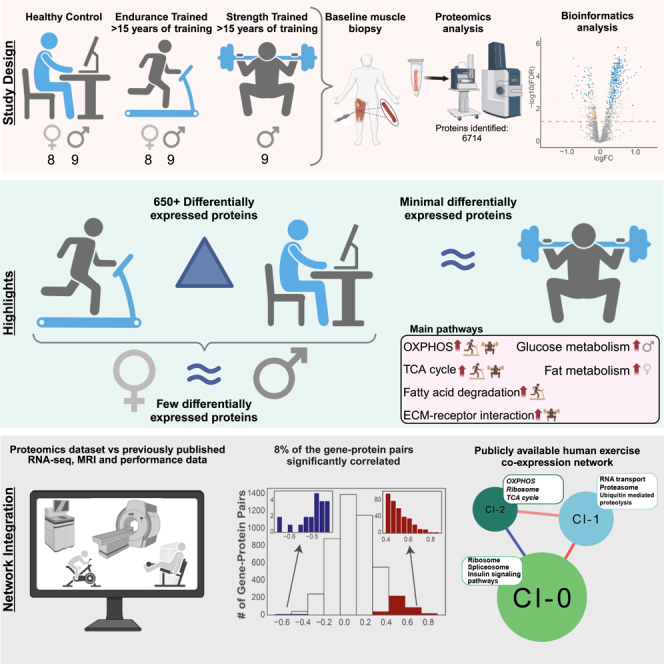

## Introduction

Regular physical activity and exercise training has tremendous health benefits, including reducing the risk of acquiring lifestyle-related diseases such as obesity and type 2 diabetes, age-related metabolic impairments, and muscle wasting.^1^^,^^2^ These benefits are associated with skeletal muscle adaptations following exercise training, including improved insulin sensitivity, increased myofiber size, and elevated oxidative capacity.^3^ Many of these beneficial adaptations can be attributed to changes in gene expression, protein abundance, and posttranslational modifications in response to exercise training.^4^^,^^5^^,^^6^^,^^7^^,^^8^ Although exercise is documented to influence gene expression and protein abundance, due to disproportionate advances in RNA sequencing technology compared with proteomics, a majority of our understanding of exercise-induced molecular changes in muscle are at the gene expression level.

Our previous work examining exercise-induced changes in skeletal muscle gene expression showed that long-term (15+ years) endurance-trained individuals had an altered resting, “baseline” (i.e., >72 h since the last exercise bout), expression of >1000 genes.^9^ However, we also showed that only 26 genes were differentially expressed at baseline between long-term strength-trained athletes and untrained controls,^9^ despite previous findings showing acute resistance training results in a significant change in expression of >600 genes.^10^ Such findings led us to hypothesize that long-term resistance training results in baseline (i.e., resting) differences in protein abundances that accrue over time instead of differences at the mRNA transcript level. However, as stated earlier, although numerous studies have investigated the skeletal muscle transcriptome following long-term exercise,^9^^,^^11^^,^^12^^,^^13^ less is known about how such alterations relate to protein abundance in highly trained humans. Previous skeletal muscle proteomic studies that have been performed focus on the effect of aging, diabetes mellitus, or on the initial adaptation process following the first weeks of exercise training,^6^^,^^14^^,^^15^^,^^16^^,^^17^^,^^18^^,^^19^ whereas studies investigating the resting muscle proteome in long-term (years) trained individuals are scarce.^20^ Understanding accumulated differences in the muscle proteome over decades of training is an important step in further elucidating the preventive medicine capabilities of long-term exercise. Additionally, advances in proteomics techniques have allowed for improvements in the number of proteins identified per sample. As such, a thorough investigation of the skeletal muscle proteome in long-term trained humans is warranted.

In addition to exercise-induced changes to skeletal muscle, it is well documented that biological sex is a significant factor that influences physiological and molecular characteristics of muscle in untrained individuals and in response to exercise.^9^^,^^21^^,^^22^^,^^23^^,^^24^ However, female research subjects are historically underrepresented in sports and exercise medicine research.^25^ Although some studies exist that examine these molecular sex differences, our knowledge of male physiology far outweighs that of females. Notably, a full proteomic characterization of sex differences in long-term trained individuals is absent from the literature, and only one study has been conducted in untrained individuals.^24^ Thus, to increase the understanding of how exercise affects the muscle proteome and imparts its health benefits across individuals, an investigation of both males and females is critical.

To address the aforementioned gaps in knowledge, the current study aimed to fully characterize the skeletal muscle proteome from long-term endurance-trained males and females, long-term strength-trained males, and age-matched healthy untrained males and females. This study design allows us to determine the protein-level alterations that accrue over decades of exercise training as well as the influence of biological sex on the muscle proteome in the untrained and trained state. An additional aim of the current study was to develop a skeletal muscle co-expression network with data from these same individuals. To accomplish these goals, we collected resting skeletal muscle biopsies from highly trained and untrained men and women. Muscle samples were then subjected to proteomic analysis, and a co-expression network was created by cross-referencing these data with transcriptomic^9^ and magnetic resonance imaging (MRI)^26^ data from the same individuals. Lastly, data in the current study were compared with published skeletal muscle proteomic datasets examining the effects of metabolic diseases and aging on muscle to investigate the preventive medicine nature of long-term exercise training.

## Results

### Study design and subject characteristics

**Figure 1:**
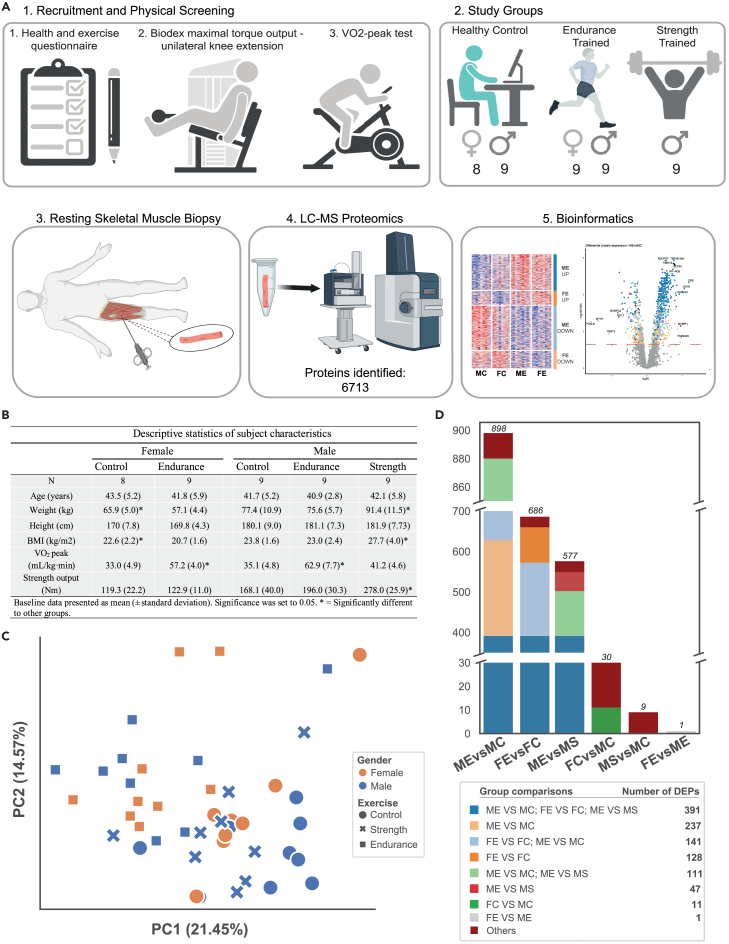
Experimental workflow and summary of differential protein expression between experimental groups (A) Flowchart of the study design. (B) Subject characteristics. (C) Principal component analysis. (D) Bar chart of statistically significant differentially expressed proteins between groups (5% false discovery rate). The color indicates how many proteins that are unique to one or more group comparisons. See also Table S1. PA, physical activity; LC-MS, liquid chromatography–mass spectrometry; PC, principal component; ME, male endurance; MC, male control; FE, female endurance; FC, female control; MS, male strength; DEP, differentially expressed proteins.

A flowchart of the study design and the subject characteristics are presented in Figures 1A and 1B and have been partially reported previously (40 of 44 subjects).^9^ In brief, a total of 44 male and female subjects were included in three different groups based on their individual exercise backgrounds and physical performance testing: (1) endurance-trained (males; ME, n = 9 and females; FE, n = 9, with at least 15 years of regular training experience), (2) strength-trained males (MS, n = 9, with at least 15 years of regular training experience), and (3) age-matched healthy untrained controls (males, MC, n = 9 and females FC, n = 8, with a self-reported history of <2 exercise bouts per week over the past 15 years). We were unable to recruit strength-trained female subjects. Per the study design, the endurance-trained individuals had a significantly higher VO_2_-peak than the strength-trained and control individuals, whereas strength-trained subjects had a higher strength output than the endurance-trained and control individuals (Figure 1B) as previously reported.^9^ Thirty-six out of fourty-four individuals underwent a whole-body MRI scan, as previously described.^26^

### Endurance-trained individuals display an altered skeletal muscle proteome signature

**Figure 2:**
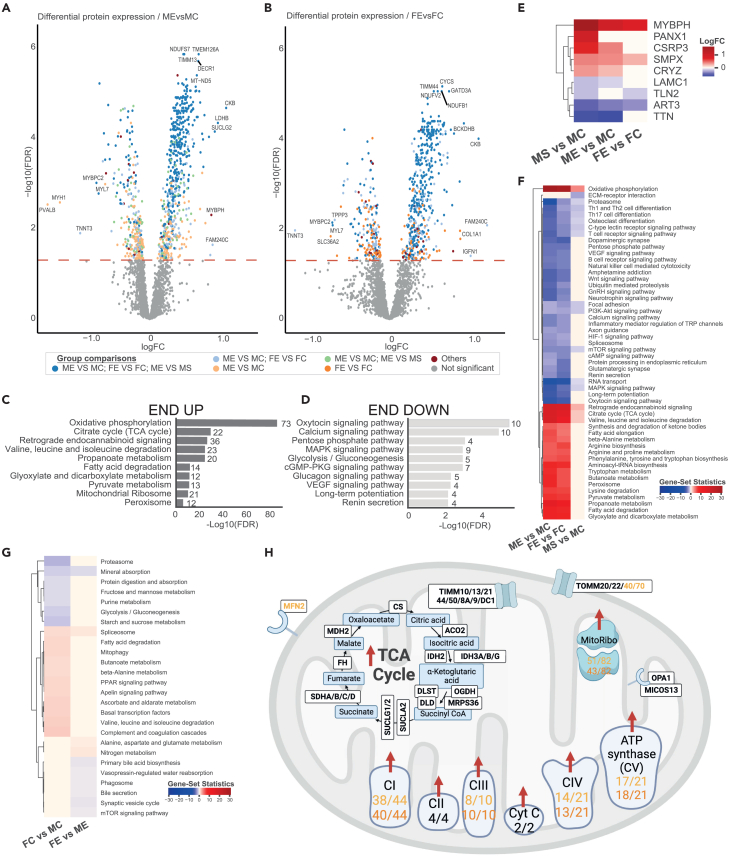
Skeletal muscle proteomic differences with long-term training and sex (A and B) Volcano plot of all DEPs between ME vs. MC and FE vs. FC, respectively. The color of each dot indicates if the protein is unique to one group comparison or shared between more group comparisons. (C and D) Overexpression analysis of the up- (C) and downregulated (D) shared DEPs in both male and female subjects versus controls using KEGG pathways. The number next to each individual bar indicates the number of DEPs within each pathway. (E) Heatmap of all DEP between MS vs. MC. Red boxes in all heatmaps indicate a significant upregulation; blue indicates a significant downregulation. (F) Gene set analysis of KEGG pathways comparing ME vs. MC, FE vs. FC, and MS vs. MC. (G) Gene set analysis of KEGG pathways comparing FC vs. MC and FE vs. ME. (H) Schematic representation of differentially expressed mitochondrial proteins with endurance training. A selection of differentially expressed mitochondrial metabolic proteins between endurance-trained males and females versus the corresponding control group. Specific proteins are displayed in white boxes. The number of differentially expressed subunits of the mitochondrial complexes (CI-V) in electron transport chain, based on MitoPathways3.0, is displayed. Yellow text = ME vs. MC. Orange text = FE vs. FC. Black text = same in both ME and FE vs. controls. Red boxes in all heatmaps indicate a significant upregulation; blue indicates a significant downregulation. LogFC, log fold change; FDR, false discovery rate. See also Figures S1–S3 and Table S1.

Liquid chromatography followed by mass spectrometry (LC-MS/MS) was performed on skeletal muscle tissue from all individuals. A total of 6,713 proteins were detected, with 4,269 proteins being expressed in at least 50% of the samples and 2,962 proteins in at least 90% of the samples. Principal-component analysis (PCA) clearly separated the samples by exercise background (Figure 1C), with the endurance group in a distinct cluster from the strength and the control groups, which partially overlapped (Figure 1C). Differential protein expression analysis revealed that 898 (65% upregulated) and 686 (71% upregulated) proteins differed between ME vs. MC and FE vs. FC, respectively (Figures 1D, 2A, and 2B; Table S1). Interestingly, of these proteins, 538 were differentially expressed in both endurance-trained males and females compared with the corresponding untrained controls (537 in the same direction—419 upregulated and 118 downregulated), which represents 60% and 78% of all differentially expressed proteins (DEPs) in ME vs. MC and FE vs. FC, respectively. Additionally, 237 and 128 proteins were found to be uniquely differentially expressed in ME vs. MC and FE vs. FC, respectively (Figure 1D; Table S1). Surprisingly, in contrast to endurance training, the male resistance-trained skeletal muscle proteome was similar to that of the untrained individuals with only nine DEPs (Figure 1D). This finding reinforces the similarity observed at the transcriptome level.^9^ Of the 577 DEPs identified in ME vs. MS, 88% (510/577) were found to be regulated in the same direction between ME vs. MS and ME vs. MC, which emphasizes the minor alteration of the baseline proteome in long-term strength-trained males. However, 47 proteins were uniquely differentially expressed between ME and MS, which were found to be primarily related to endoplasmic reticulum calcium ion homeostasis (GO: 0032469) and myofibril assembly (GO: 0030239) (Table S1). Lastly, our analysis revealed that sex differences diminished from 30 DEPs found between the male and female controls to just 1 DEP between FE and ME—PUDP, a protein with no known biological function (Figure 1D; Table S1; Figure S1).

To explore the function of the proteins that were differentially expressed in endurance-trained muscle, i.e., proteins found in both FE and ME compared with the corresponding control group, an overrepresentation analysis of the DEPs was performed (Figures 2C and 2D). Proteins higher in endurance-trained individuals were related to pathways involved in oxidative phosphorylation (OXPHOS), the tricarboxylic acid (TCA) cycle, fatty acid degradation, amino acid metabolism, and the mitochondrial ribosome (Figure 2C). The downregulated proteins in the endurance groups were related to glucose metabolism and calcium/MAPK signaling pathways (Figure 2D). These endurance-training adaptations reflect expected oxidative energy turnover capacity and agree with previous studies at the transcriptomic and proteomic levels.^9^^,^^16^^,^^18^ Lastly, as many DEPs were shared between ME and FE compared with their respective control groups, we investigated the number of DEPs in a sex-independent comparison of all endurance-trained individuals and all control subjects. A total of 1,169 DEPs were identified, with 199 being uniquely expressed (i.e., DEPs found in this analysis were not found in either ME vs. MC or in FE vs. FC, Table S1). However, pathway analysis revealed that the function of these proteins is largely the same as the sex-specific comparisons.

Only a few proteins were found to be differentially regulated between the resistance-trained and untrained male subjects (Figure 2E). Among these proteins, the upregulation of cysteine- and glycine-rich protein 3 (CSRP3) and Pannexin 1 (PANX1) is particularly interesting as they have important roles in skeletal muscle myogenesis^27^ and proliferation/differentiation of skeletal muscle myoblasts, respectively.^28^ CSRP3 has previously been shown to be induced in skeletal muscle at 6 and 24 h following acute eccentric exercise.^27^ Additionally, knockout models have shown that the absence of CSRP3 mRNA expression inhibits terminal differentiation of skeletal muscle cells,^27^ resulting in impaired glucose tolerance and systemic insulin sensitivity in mice, and it is suggested to play a role in obesity-induced diabetes and inflammation.^29^ Thus, even though strength training does not appear to shift global baseline protein or gene expression, individual proteins with important roles in muscle hypertrophy and maintaining a healthy metabolism were identified following long-term strength training. It should be noted, however, that acute changes in gene/protein expression have previously been shown to contribute strongly to the characteristic hypertrophic phenotype seen in resistance-trained individuals.^4^^,^^10^^,^^30^ Together with the current study, this suggests that acute changes in gene/protein expression with resistance training serve to stimulate hypertrophic pathways that ultimately result in increases in muscle mass, but do not dramatically change the baseline expression of genes/proteins. As such, we suggest that the increased muscle mass in the MS group is accompanied by a proportional increase and unaltered relative abundance of myofibrillar and ribosomal proteins.

To deepen our understanding of the functional implications of the differential proteome, we performed a gene set analysis (GSA) of all identified proteins using the reporter method.^31^ Similarly to the overrepresentation analysis of DEPs presented in Figure 2C, the pathways most elevated by long-term training were related to OXPHOS, TCA cycle, fatty acid degradation, and branched chain amino acid degradation (BCAA—i.e., valine, leucine, and isoleucine) compared with controls although to a higher extent in the endurance groups compared with the strength group (Figure 2F). Downregulated pathways found in both endurance- and strength-trained individuals were mainly related to the proteasome and immune system pathways (Figure 2F). Previous research has shown an upregulation of the proteasome pathway following acute exercise^32^ but also at baseline in insulin-resistant skeletal muscle with aging and in individuals with T2D.^17^^,^^19^ It has been suggested that a single bout of exercise acutely upregulates the proteasome pathway to mediate muscle remodeling during muscle adaptations but that a long-term baseline upregulation could lead to excessive degradation of critical regulatory proteins.^32^ These data suggest that regular exercise training can lead to metabolically protective decreases in protein degradation and, thus, a sustained maintenance of muscle mass in trained individuals.

In terms of sex differences, the low number of DEPs found comparing both endurance-trained (n = 1) and untrained (n = 30) females and males was surprising considering the substantial sex differences found at the transcriptomic level in these same individuals^9^ and in a previous study.^22^ Despite the small numbers of DEPs, GSA suggested several functional differences between the sexes (Figure 2G). Compared with untrained males, untrained females displayed higher levels of fatty acid degradation, mitophagy, and several amino-acid-related pathways, whereas pathways related to carbohydrate metabolism and the proteasome were lower (Figure 2G). However, none of these pathways were significantly enriched in endurance-trained females compared with males (Figure 2G), indicating a more “functionally similar” phenotype between the sexes that could be a result of years of regular training, a finding that echoes the transcriptomic data from these same individuals.^9^

Given that most of the enriched pathways in the overrepresentation analysis were associated with mitochondrial functions, we moved on to investigate the specific mitochondrial- and non-mitochondrial-related adaptations to endurance training in skeletal muscle. To accomplish this, known mitochondrial proteins, based on MitoCarta 3.0 and the Integrated Mitochondrial Protein Index, were separated from the DEPs altered with endurance training.^33^^,^^34^ This analysis showed that 92% of the shared proteins with higher expression in both ME and FE were known mitochondrial proteins, whereas only five of the proteins with lower expression were classified as mitochondrial (Figure S2; Table S1). Additionally, a majority of the DEPs with the highest fold change and lowest false discovery rate were known mitochondrial proteins (Figure S2; Table S1). When investigating mitochondrial DEPs, subunits from all mitochondrial complexes in the electron transport chain, proteins in the TCA cycle, translocases of the outer and inner mitochondrial membranes (TOMs and TIMs), and mitochondrial dynamin-like GTPase (OPA1) were all increased in both ME and FE compared with the corresponding controls (Figure 2H). This shows that proteins related to mitochondrial bioenergetics, biogenesis, fusion, and proteins responsible for the translocation of cytosolically synthesized mitochondrial preproteins^35^^,^^36^ were more abundant in endurance-trained individuals. To get a better understanding of the function of the mitochondrial DEPs, a gene set enrichment analysis using MitoPathways3.0^33^ was performed for all experimental groups. As expected, both ME and FE displayed a significant enrichment of all the main high-level pathway categories, including mitochondrial central dogma, protein import sorting and homeostasis, OXPHOS, metabolism, small molecule transport, signaling, and mitochondrial dynamics and surveillance (Figure S3). Although only nine DEPs were found between MS and MC, the MitoPathways gene set enrichment analysis revealed several mitochondrial pathways to be significantly upregulated with resistance training, albeit to a lesser extent compared with endurance training (Figure S3). These findings agree with the literature that shows that resistance training is accompanied with mitochondrial adaptation.^37^

In terms of the 40 non-mitochondrial proteins that increased in the endurance groups, overrepresentation analysis identified no significantly enriched pathways among these upregulated proteins. As no significant pathways were identified, we investigated upregulated non-mitochondrial proteins with previously known metabolic functions in the endurance-trained versus untrained subjects. Of specific interest among these proteins with increased expression in the endurance groups, creatine kinase B, malate dehydrogenase 1, fatty-acid-binding protein 3, SLC16A1, and lactate dehydrogenase B (LDHB) were found, which all have important functions in various metabolic processes.^38^^,^^39^^,^^40^^,^^41^^,^^42^ Specifically, previous research has demonstrated an increase of LDHB enzyme activity and gene expression in humans in response to endurance exercise.^20^^,^^38^^,^^43^ Furthermore, transgenic mice with increased running capacity have an increased *Ldhb/Ldha* gene expression ratio, which was found to be associated with improved insulin sensitivity and increased protein levels of GLUT4.^44^ Furthermore, the elevated *Ldhb/Ldha* ratio and GLUT4 protein levels in these mice resulted in increased glucose uptake, glycogen storage, and mitochondrial pyruvate oxidation. Similarly, in the current study, ME displayed a greater SLC2A4 (also known as GLUT4) abundance compared with MC, whereas a non-significant increase in FE vs. FC was present (FDR = 0.14). These findings suggest that endurance-training induced changes in non-mitochondrial protein abundance influence skeletal muscle metabolic processes, particularly in optimizing the ATP-generating pathways of glucose and fat oxidation. Taken together, even though these alterations are related to non-mitochondrial proteins, they all serve to support the mitochondria by optimizing ATP generation.

### Correlation between transcriptome and proteome in human skeletal muscle

**Figure 3:**
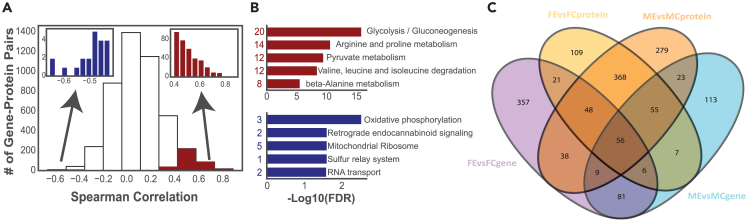
Correlation between global gene and protein analysis (A) Density plot of the correlation (Spearman rank correlation) of all gene-protein pairs, with the statistically significant (5% false discovery rate, FDR) pairs highlighted in red (upregulated) and blue (downregulated). (B) Functional analysis of the significantly correlated pairs, based on (A). The number of gene-protein pairs within each pathway is included next to each pathway. (C) A Venn diagram of all differentially expressed proteins and the differentially expressed genes that were detected also at the protein level, between both endurance groups and the corresponding controls. See also Figure S4 and Table S2.

When comparing the current study with our previously published transcriptomics data, protein and gene differential expression patterns were similar across all comparisons, with the largest differences found with endurance training, sex differences decreasing with endurance training, and minimal differences with resistance training.^9^ Functional association of the differential transcriptome and proteome was also similar across these same comparisons. Thus, we found that the skeletal muscle transcriptomic and proteomic analyses are largely concordant and are both valid tools in examining muscle physiology. As there is a long-standing debate in the field of molecular biology regarding the presumed direct relationship between changes in mRNA expression and protein abundance,^45^ we performed ranked Spearman correlation (*r*_*s*_) analysis based on all detected proteins and the corresponding genes to understand the direct gene-to-protein relationship between our transcriptomic and proteomic data from these individuals. Unlike the similarities seen at the pathway level, only 8.4% of the gene-protein pairs were significantly correlated, of which 95% were positively correlated (Figure 3A; Table S2), which is in line with previous data.^46^ Functional analysis of the significantly correlating gene-protein pairs showed that positively correlated pairs were involved in the glycolysis, amino acid metabolism, and pyruvate metabolism pathways (Figure 3B). Previous work also showed concordant RNA and protein expression of insulin and glucagon, which are regulated by blood glucose levels.^46^ The negatively correlated gene-protein pairs displayed no significantly enriched pathways, but their functions were associated with oxidative phosphorylation, the mitochondrial ribosome, and RNA transport (Figure 3B). Interestingly, when investigating individual gene-protein pairs, 8 of the 13 mitochondrial-encoded proteins were found to be significantly positively correlated (Table S2), which could be related to mtRNA posttranscriptional modifications that serve to stabilize mtRNA and, thus, increase its half-life.^47^ Thus, although the comparisons between our proteomic and transcriptomic data demonstrate similar numbers of differentially regulated genes/proteins as well as their overarching functions, the one-to-one correlations between these data types are poor. Therefore, researchers should not extrapolate baseline gene expression data to protein expression.

As the proteomic data are relative values to the pooled samples, we were unable to perform a correlation analysis based on absolute expression values. Therefore, we moved on to investigate the overlap of DEPs and differentially expressed genes (DEGs) in the respective analyses with an emphasis on the effects of endurance training as this drives a majority of differential expression in our datasets. Firstly, all DEGs not detected at the protein level, and vice versa, were removed (Figures S4A and S4B) from this analysis to represent identifiable correlations between DEGs and DEPs with endurance training (Figure 3C). In the endurance-trained individuals, 2,393 DEGs and 1,039 DEPs were found to be differentially regulated (either in FE vs. FC, ME vs. MC, or both). However, 1,579 (66%) of the DEGs were not detected at the protein level in >50% of the samples, and 20 DEPs were not detected in the RNA sequencing analysis (Figures S4A and S4B). The removal of these gene-protein pairs resulted in 814 DEGs, and 1,019 DEPs remaining to be compared. Of these, 263 endurance-induced pairs were differentially expressed at both the gene and protein levels (238/263 in the same direction; Figure 3C; Table S2). Further analysis of these 263 gene-protein pairs showed that 56 were common DEPs and DEGs in both FE and ME (55/56 in the same direction), 48 were DEPs in FE and ME but only found to be DEGs in FE (41/48 in the same direction), whereas 55 were DEPs in FE and ME but were only DEGs in ME (all in the same direction; Figure 3C; Table S2). A small number of proteins and genes were identified as differentially regulated in only one sex; 21 were exclusively differential in FE vs. FC (18/21 in the same direction) and 23 in ME vs. MC (21/23 in the same direction; Figure 3C; Table S2).

Notably, 75% of the 756 differential proteins in endurance-trained males and females were not differentially expressed at the gene level (Figure 3C). One potential explanation for this discrepancy is that at the time of biopsy collection (72-h post exercise), mRNA transcripts responsive to acute exercise have returned to baseline while the corresponding proteins remain elevated. To investigate this hypothesis further, we used data from our laboratory^48^ investigating global gene expression following a 30 min bout of endurance exercise in endurance-trained and untrained males. Of the 647 DEPs identified without a corresponding DEG in endurance-trained males, we found that 105 (15.8%) were acutely regulated within 3 h after an acute cycling bout in either endurance-trained or untrained males (Figure S4F). It is possible that a fraction of the remaining 558 DEPs were acutely regulated later than 3 h, as it has previously been shown that gene expression generally peaks ∼4–8 h following an acute bout of exercise.^49^ As gene expression often precedes changes in protein abundance,^50^ a time series of biopsies, from immediately after to 72 h after exercise, would improve our understanding of the interplay between gene-protein pairs, but this was beyond the scope of the current study. Thus, there is a need for a temporal multi-omics approach following acute exercise to thoroughly understand how transcriptomic and proteomic networks interact to induce the adaptations accompanying exercise training.

### Network analysis reveals central analytes important to exercise adaptation related to physiological data

**Figure 4:**
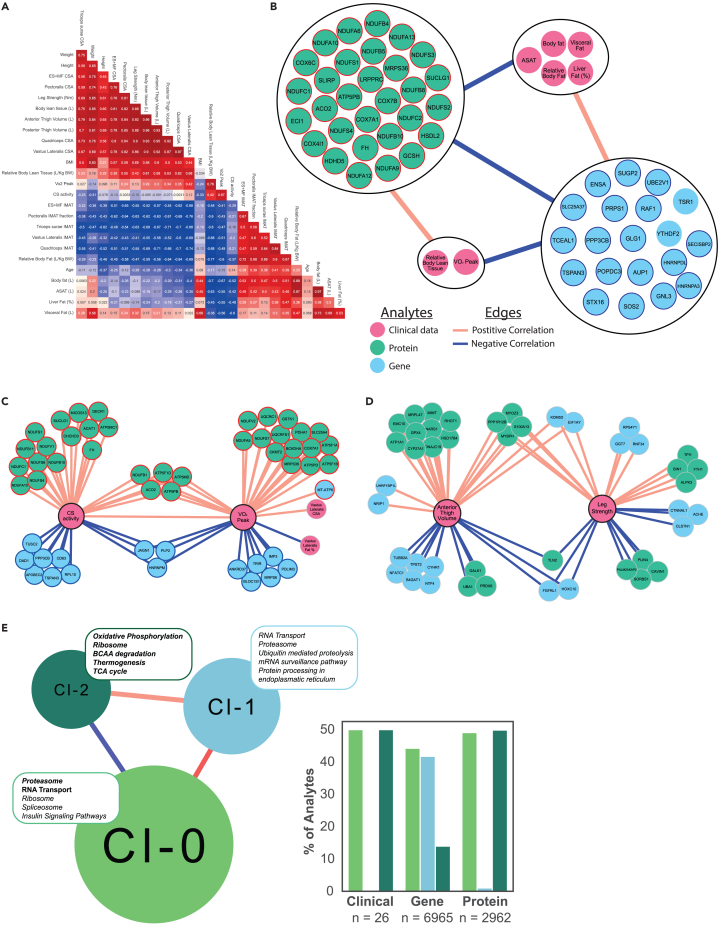
Network analysis of global gene and protein expression data (A) Correlation matrix of “clinical data,” including subject characteristics, MRI data, and exercise performance data. (B) Centrality analysis of the most central analytes (proteins in green, genes in blue, and clinical outcome measures in pink circles). (C) Sub-network of the strongest correlating differentially expressed proteins and genes compared with maximal oxygen uptake (VO2-peak) and citrate synthase activity (CS activity). The circle border color in B & C indicates the direction of gene and protein expression, blue = significantly downregulated, red = significantly upregulated (5% false discovery rate) in endurance versus control. (D) Sub-network of the strongest correlating proteins and genes compared with anterior thigh volume and leg strength. (E) The three main clusters found from community detection of all genes, proteins, and clinical data and the proportion of analytes within each cluster. The five most enriched pathways based on analytes within each cluster are displayed, bold = pathways from proteins, italic = pathways from genes, bold italic = enriched in both proteins and genes within the cluster. Red lines = significant positive correlation. Blue lines = significant negative correlation. See also Figures S3 and S4.

In order to elucidate functional relationships between our omics data and clinical data (Figure 4A), we generated a co-expression network combining these data. As expected, several parameters associated with beneficial health outcomes were positively correlated, whereas parameters associated with negative health outcomes were negatively correlated with our performance metrics (Figure 4A). To identify the drivers of this co-expression network, a centrality analysis of the most central genes, proteins, and clinical data was generated (Figure 4B). The centrality analysis highlights the analytes with the most connections (i.e., analytes with most significant associations with other functionally related genes or proteins) with other analytes in the network (degree of centrality is proportional to the importance of the analyte in the co-expression network). It is hypothesized that a removal of these most central analytes (e.g., in a transgenic knockout model or an *in vitro* knockdown experiment) would lead to a significant alteration of phenotypes.^51^ Indeed, an investigation of the most central proteins identified in Figure 4B using the International Mouse Phenotyping Consortium database demonstrated metabolic impairments in mice heterozygous for some of these central proteins: NDUFA10 (increased body fat), NDUFS1 (increased circulating lipase, decreased activity), ACO2 (increased circulating cholesterol), NDUFS3 (increased circulating glucose levels, decreased circulating HDL cholesterol levels), and MRPS36 (decreased lean body mass, increased total body fat amount).^52^ Thus, the most central genes and proteins in the network are suggested to drive the clinical data with which they are significantly correlated. The analysis shows that the most central proteins were all positively correlated with “favorable” clinical data, such as a higher VO_2_-peak and relative lean body tissue (Figure 4B). In contrast, the most central genes were all negatively correlated with these same “favorable” data, whereas they were positively correlated with “unfavorable” clinical parameters such as higher relative body fat, abdominal subcutaneous adipose tissue (ASAT), visceral fat, and liver fat % (Figure 4B). Among the most central proteins were subunits of mitochondrial complex I (e.g., NDUFBs, NDUFAs, and NDUFSs) and IV (COX6C, COX4I1, COX7A1, and COX7B) as well as proteins that play an essential role in the TCA cycle (FH and ACO2) (Figure 4B). The most central genes were functionally related to proteasome subunits (PSMA3, PSMC5, and PSMD6) and B and T cell receptor signaling pathways (PPP3CB, RAF1, SOS2, and RHOA) (Figure 4B; Table S3).

Subsequently, in order to identify the main drivers of exercise performance, we created two “exercise networks” associating the analytes most strongly correlating with endurance (VO_2_-peak and citrate synthase [CS] activity) and resistance (leg strength and anterior thigh volume) exercise performance outcomes (Figures 4C and 4D). Five proteins (ATP5PB, ATP5MD, ATP5F1D, ACO2, and NDUFB1) were strongly correlated with CS activity and VO_2_-peak and were also found to be significantly upregulated in both ME and FE compared with controls. Of these, three are ATP synthases in mitochondrial complex V (ATP5PB, ATP5MD, ATP5F1D), NDUFB1 is a subunit of complex I, and ACO2 catalyzes the isomerization of citrate. Thus, all five of these central proteins have important functions for the generation of ATP during oxidative phosphorylation and the TCA cycle.^53^ Of the top 50 most positively correlated analytes to either CS activity or VO_2_-peak, 48 and 49 respectively, were proteins, all of which were known mitochondrial proteins (Table S4). This further confirms the importance of mitochondrial adaptations for athletic performance in endurance sports. Also, these data largely confirm that protein expression levels are more closely associated with phenotypic adaptations to endurance training compared with gene expression levels in our subjects.

In a sub-network highlighting genes and proteins related to anterior thigh volume and leg strength, both were found to be positively correlated with skeletal muscle Z-line protein (MYOZ3^54^), cytosolic calcium-binding protein (S100A13^55^), myosin-binding protein (MYBPH^36^), and a myosin phosphatase (PPP1R12B^36^), with the latter two being part of the regulation of muscle contraction (GO:0006937) Gene Ontology pathway. These correlations further demonstrate the importance of muscle structural proteins as well as calcium-binding proteins in establishing hypertrophy and muscle strength.^56^^,^^57^^,^^58^ Additional correlations between proteins, genes, and clinical data in this network can be investigated on the publicly available database iNetModels 2.0^59^ under “Multi-Omics Network” → “Study-specific Networks” → “Long-Term Exercise (Emanuelsson et al. 2023)". This interactive database can be used by other investigators for hypothesis generation of candidate genes, enhancing understanding of specific analyte-analyte interactions, and determining how specific analytes correlate with clinical data such as specific MRI measures.

To better understand the entire analyte network, we performed Leiden community detection analysis^60^ to identify the main clusters of analytes and detected three main clusters (Figure 4E). Additionally, we investigated the main functions of each cluster as well as the associations between the three identified clusters. All of the top 50 most central proteins (identified in Figure 4B) were found in Cluster 2 and showed a high association with metabolic adaptations and clinical parameters typically seen with endurance training (Figure 4E; Table S3). Additionally, the top upregulated pathways based on both proteins and genes within this cluster were related to mitochondrial and metabolic processes. Clusters 0 and 1 contained the top 50 most central genes (in Figure 4B), were dominated by analytes upregulated in the control versus endurance groups, and were positively correlated with each other but negatively correlated with Cluster 2 (Figure 4E; Table S3). Clusters 0 and 1 primarily appear to be dominated by processes related to the regulation of the central dogma (i.e., processes related to RNA and protein production, processing, and degradation). Specifically, analytes in Cluster 0 were related to the proteasome and spliceosome pathways, RNA transport, and ribosome-related gene ontologies, whereas Cluster 1 mainly included genes functionally involved in RNA transport, protein degradation, and mRNA processing (Figure 4E). Taken together, this multi-omics network provides novel insights into how genes and proteins are associated with long-term exercise training adaptations and clinical MRI data. Specifically, we identified the most important genes and proteins, i.e., the analytes with most interactions with other analytes, with a centrality analysis. Furthermore, these analytes were correlated with exercise performance data and MRI data that add a layer of clinically functional relevance, which is typically absent from conventional differential gene/protein analyses. Next, our community detection analysis displays the main functions of three identified clusters, which compliments and further contextualizes the functional analysis of the DEPs identified in this study (in Figure 2). Furthermore, the clusters provide future references on which network aspect to focus on for studying specific subsystems or pathways associated with molecular response of skeletal muscle to long-term exercise. These findings further highlight specific genes, proteins, and pathways that are important for the health benefits related to regular exercise training throughout life.

### Positive correlation between endurance-trained and young skeletal muscle proteome

**Figure 5:**
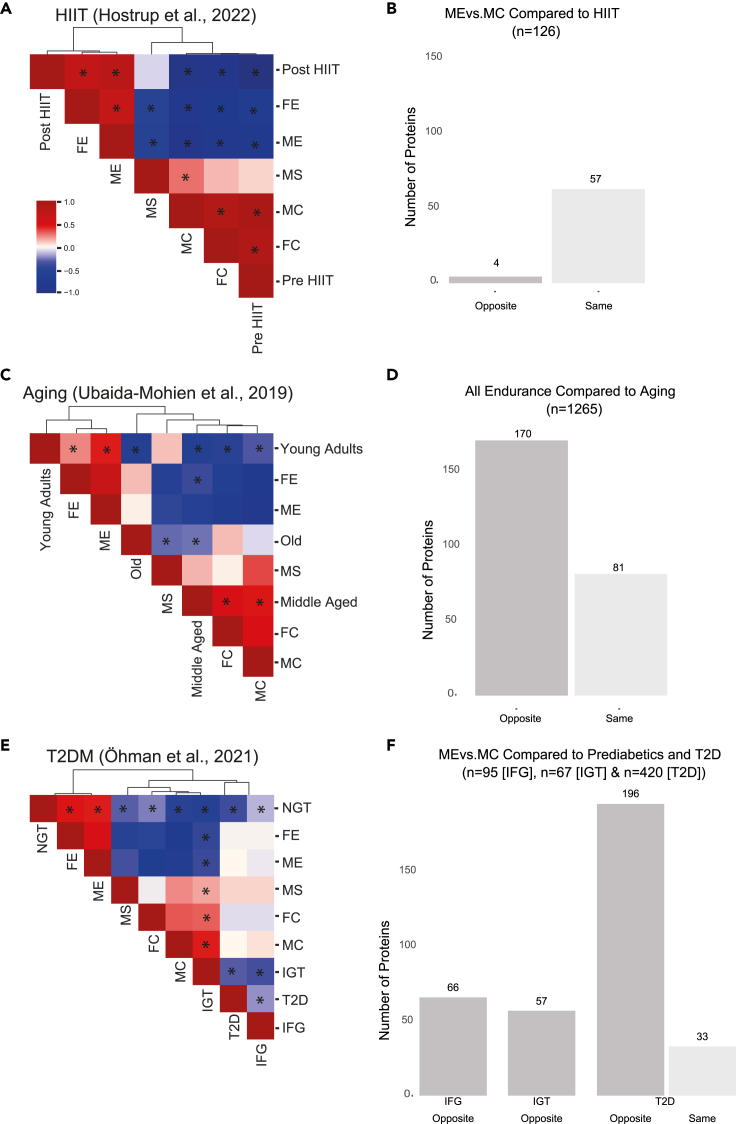
Correlations between trained muscle proteomes from trained males and individuals with various metabolic conditions (A–F) Correlations of protein expression of DEPs in the current study with (A and B) an HIIT intervention in young males, (C and D) aging including young adults (20–49 years), middle-aged (50–64 years) and old adults (65+ yrs), and (E and F) a type 2 diabetes (T2D) study, including men with normal glucose tolerance (NGT), impaired fasting glucose (IFG), impaired glucose tolerance (IGT), and T2D. (B), (D), and (F) represent comparisons of directionality of the differentially expressed proteins in the current compared with the external datasets in (A, C, and E), respectively. For clarity, significant correlations between study groups from the current study are only depicted in 5A. ∗ = FDR <0.05. See also Figure S5.

To further contextualize our results, we compared our data and proteomics data from individuals with various metabolic impairments as this could shed light on the proteins that impart the preventive medicine capabilities of long-term exercise. To do so, we cross-referenced our proteomic data with previously published skeletal muscle proteomic datasets investigating the influence of aging^19^ and type 2 diabetes (T2D)^61^ on the skeletal muscle proteome (Figure 5). Additionally, we included a short-term (5-week) HIIT intervention study as a “positive control” for the endurance groups.^16^ First, we correlated the relative abundance of shared DEPs between the current study and external datasets, in all study groups. Second, as few DEPs were identified with sex or strength training, we investigated the directionality of the shared DEPs in the long-term endurance-trained groups with the same external datasets.

As expected, a strong positive correlation was found between the skeletal muscle proteome of both ME and FE compared with the HIIT-trained individuals after 5 weeks of training (Figure 5A). Of the 61 shared DEPs, 57 were in the same direction, whereas 4 were in the opposite direction between the studies, suggesting that the DEPs found in our endurance-trained versus control groups occurred due to regular endurance training (Figure 5B). Additionally, functional analyses revealed that similar pathways, although to a smaller magnitude, were enriched after 5 weeks of training^16^ as after >15 years of training. This suggests that even after a short (5-week) training program, high-intensity endurance training is sufficient to initiate adaptations related to the TCA cycle and oxidative phosphorylation that accompany life-long training. However, as seen in the current study, longer periods (years) of training lead to an increased number of DEPs involved in these pathways and mitochondrial complexes, likely due to a greater aerobic capacity.

Following confirmation of the endurance adaptations, we correlated our data with data from the studies examining aging and T2D. A positive correlation was found between our untrained control groups (33–52 years old) and healthy middle-aged (50–64 years old) individuals, whereas these same individuals were negatively correlated with young adults (20–49 years old; Figure 5C). Interestingly, the endurance-trained individuals were negatively correlated with the middle-aged cohort, but positively correlated with the young adults. MS was negatively correlated with the older cohort (65+ years old), whereas neither of the control groups nor the endurance groups were significantly correlated with the older cohort. Next, we compared the “age-associated” DEPs (n = 1265)^19^ and DEPs found with long-term endurance training. A total of 251 DEPs were shared between the studies, of which 170 were oppositely regulated (Figure 5D). Of these 251, 44% were mitochondrial proteins, mainly related to thermogenesis and OXPHOS (Table S5). Collectively, these findings suggest that an endurance-trained proteome is more similar to the protein signature of skeletal muscle in young individuals, demonstrating the ability for endurance training to maintain a healthier phenotype.^6^^,^^62^

When compared with the T2D study (including males with: normal glucose tolerance [NGT], impaired fasting glucose [IFG], impaired glucose tolerance [IGT], and T2D), we found a positive correlation between our endurance-trained groups and the NGT but a negative correlation between IGT and the endurance groups (Figure 5E). However, our control groups and MS were positively correlated with the IGT group (Figure 5E). Although no statistically significant correlations were found between the T2D or IFG and the endurance-trained or untrained control groups, comparisons of DEPs between the studies showed that several were oppositely expressed in ME versus IFG (n = 66), IGT (n = 57), and T2D (n = 196; Figure 5F; Table S5). Interestingly, 88%–97% (IFG = 97%; IGT = 88%; T2D = 89%) of the oppositely regulated proteins were mitochondrial proteins (Table S5), including subunits of all mitochondrial complexes in IGT and T2D and all but complex II in IFG. Additionally, the insulin- and contraction-regulated glucose transporter SLC2A4 (GLUT4), which is important for glucose removal from circulation to the skeletal muscle, was oppositely regulated between ME and T2D but not in IFG or IGT. The dysregulation of mitochondrial proteins in T2D, particularly related to OXPHOS, has previously been described,^14^^,^^17^^,^^61^ but this study demonstrates the ability of long-term endurance training to increase these same mitochondrial proteins and pathways that are implicated in T2D and prediabetes.^63^ These data, together with previous reports,^6^^,^^64^ further emphasize the potential of regular exercise training to enhance skeletal muscle insulin sensitivity.

Overall, the comparative analyses presented here suggest that regular training, and endurance training in particular, positively affects the abundance of proteins that are misregulated in various detrimental metabolic conditions. The general role of these proteins is related to mitochondrial function, and, specifically, oxidative phosphorylation. Thus, we suggest regular endurance training to have the potential to eliminate or, at least, attenuate the aging- and T2D-associated loss in mitochondrial protein expression that has been shown to accompany both aging and T2D.^6^^,^^61^^,^^62^ However, longitudinal studies that are beyond the scope of the current study would be needed to better understand how regular exercise training may influence the abundance of proteins negatively affected by metabolic diseases and aging to more definitively demonstrate these links.

## Discussion

This paper presents a full skeletal muscle proteomic characterization of long-term trained humans, which allows us to investigate the proteomic differences that accompany long-term trained and untrained individuals as well as the presence of potential sex differences. Overall, this study showed a significantly altered skeletal muscle proteome in highly endurance-trained males and females, but not in strength-trained males, compared with untrained controls. The DEPs found in both endurance-trained males and females showed that over 90% of the proteins with higher expression in trained individuals were mitochondria-related. The functions of these proteins were primarily related to an upregulation of oxidative phosphorylation, the TCA cycle, and fatty acid degradation pathways. Interestingly, only a few proteomic sex differences were found between our untrained research subjects, but the sex differences that did exist were essentially eliminated in the endurance-trained subjects. This suggests that long-term endurance training results in a convergence upon a skeletal muscle proteomic signature that is largely independent of sex assigned at birth. In contrast to endurance training, only nine proteins were differentially expressed between strength-trained and untrained males, which is in line with previous findings on the transcriptomic level.^9^ Although strength training does not appear to shift baseline gene/protein expression, it should be noted that this does not mean that resistance training does not have substantial health benefits. Indeed, increased muscle mass and muscular strength associated with strength training is beneficial in maintaining a healthy metabolism, bone density, and motor control as humans age.^6^^,^^65^^,^^66^

There is a long-standing debate in the field of molecular biology regarding the presumed direct relationship between changes in mRNA expression and protein abundance.^45^ Previous research has suggested that the genome-wide relationship between mRNA levels and proteins is relatively poor, with mRNA levels explaining around 40% of the protein expression levels.^67^ It should, however, be noted that the range of the gene-protein expression R^2^-values in multi-cellular organisms ranges from 0.09 to 0.46.^68^ Additionally, RNA expression findings from transcriptomic studies are assumed to be representative of how cells/tissues are responding at the protein level to a variety of stimuli, but the one-to-one correlation between RNA expression and the corresponding protein abundances do not necessarily support this assumption.^46^ In line with these previous studies, our correlation analysis between proteomic and transcriptomic data in muscle revealed a poor one-to-one relationship between genes and proteins. However, functionally speaking, both proteomic and transcriptomic analyses yielded similar results. The discrepancy between proteomic and transcriptomic data are further highlighted by the fact that 67.7% of all endurance-induced DEGs were not found to be DEPs, which could be attributed to the regulation of protein translation. Specifically, previous studies have shown that protein translation is inhibited by noncoding RNAs, suggesting a plausible mechanism behind the identification of differentially regulated genes at the mRNA level in the absence of differences at the protein level.^69^^,^^70^ Additionally, functional analysis of the DEGs that were not found to be DEPs revealed an overrepresentation of ribosomal protein genes (RPGs). As previous reports suggest, translational activation of RPGs is thought to be a mechanism by which cells rapidly regulate these proteins in order to circumvent the slower transcriptional activation process of these genes.^71^ As such, these RPGs would be present at the mRNA level but not at the protein level, which could further explain some of the differences we see in DEGs versus DEPs in the present study. Additionally, as various skeletal muscle proteins display different half-lives,^72^ proteins with shorter half-lives require more mRNA bursts or continuous mRNA expression to sustain/increase protein expression, which could also explain some of the disparities seen in these data. Lastly, because mass spectrometry is less sensitive compared with RNA sequencing, some of the differences here could be attributed to technical limitations of the experimental setup, especially for low-abundance proteins.^46^

Recent studies have started to integrate different omics data into co-expression networks to better understand various diseases.^51^^,^^73^ This development can help researchers understand the variations between healthy and diseased tissues and can lead to optimized patient treatment strategies.^51^ Furthermore, by including data from highly trained individuals in these networks, which have a reduced risk for several lifestyle-related disorders,^2^ the potential to optimize and personalize treatment increases further. Specifically, the preventive-medicine capabilities of long-term training can be elucidated from the co-expression network and comparative literature analysis in the current study, which revealed key proteins and pathways related to the health benefits of exercise. For instance, our analysis methods identified fumarate hydratase (FH), a key component of the TCA cycle where it converts fumarate to L-malate, as a protein of high importance in the adaptation to long-term exercise. Not only was FH found to be increased in both ME and FE groups compared with controls, but our network and co-expression analyses found that it was among the top 0.5% most central proteins and was positively correlated with both VO_2_-peak and CS activity. Furthermore, our comparative analysis identified FH as an oppositely regulated protein when comparing our exercise training dataset with the aging and T2D studies. Taken together, our current analysis shows that increased FH levels with endurance training could attenuate metabolic-dysfunctions-associated mitochondrial impairments. To the authors’ best knowledge, this is the first study to generate a multi-omics exercise network of well-characterized females and males, which integrates proteomic, transcriptomic, MRI, and exercise performance data, and is publicly available in a user-friendly interactive database. This network provides insights into the functional relationships of genes and proteins associated with long-term training adaptations such as exercise performance and clinical MRI data. Additionally, the database can be used by other investigators for hypothesis generation of candidate genes, enhance our understanding of specific analyte-analyte interactions, and investigate how specific analytes dictate clinical data, such as specific MRI measures.

### Limitations of the study

The cross-sectional design of this study means we cannot rule out the possibility that differences found between trained and untrained individuals might be present due to factors (i.e., genetic and/or dietary variations) other than exercise training background. To address this, we cross-referenced our data with a study examining the influence of a 5-week HIIT intervention on the skeletal muscle proteome.^16^ We found that roughly 50% of the proteins with an altered abundance following HIIT were also differentially expressed in the same direction in our male endurance versus control subjects. Another limitation of the current study is the fact that we were unable to recruit highly strength-trained women. Future studies are needed to better understand the muscle adaptations of both sexes, with a special focus on female athletes.^25^ Lastly, it should be noted that the correlations between our cohorts and other publicly available datasets are not direct comparisons. Instead, the correlations are calculated from the fold change relative to the pooled samples of a given protein between the intervention group and the control group. This is a limitation because it makes direct quantitative correlations impossible; however, this type of comparison is still appropriate as it improves our understanding of the preventive-medicine capabilities of long-term training.

## STAR★Methods

### Key resources table

**Table:** 

REAGENT or RESOURCE	SOURCE	IDENTIFIER
**Deposited data**		

Proteomics data	This manuscript	PRIDE: PXD044445
RNAseq data	Chapman et al.^9^	EGA: S00001004367
MRI data	Emanuelsson et al.^26^	PMID: 35854646
Proteomics data	Hostrup et al.^16^	PRIDE: PXD023084
Proteomics data	Ubaida-Mohien et al.^19^	PRIDE: PXD011967
Proteomics data	Öhman et al.^61^	PMID: 34235411

**Software and algorithms**		

Original code	This manuscript	https://doi.org/10.5281/zenodo.8436464
Network data	This manuscript	iNetModels: https://bit.ly/iNetModels_LongTerm-Exercise
PIANO	Väremo et al.^31^	RRID: SCR_003200
GSEAPY	Fang et al. ^74^	https://doi.org/10.1093/bioinformatics/btac757
KEGG	Kanehisa and Goto^75^	RRID: SCR_012773
MitoCarta3.0	Rath et al. ^33^	RRID: SCR_018165
FGSEA	Korotkevich et al.^76^	RRID: SCR_020938

### Resource availability

#### Lead contact

Further information and requests for resources and reagents should be directed to and will be fulfilled by the Lead Contact, Mark Chapman (markchapman@sandiego.edu; mark.chapman@ki.se).

#### Materials availability

This study did not generate new unique reagents.

#### Data and code availability

• The mass spectrometry proteomics data have been deposited at ProteomeXchange Consortium via the PRIDE^77^ partner repository and are publicly available as of the date of publication. Data from two^16^^,^^19^ of the three previously published studies are accessible through ProteomeXchange and the last dataset^61^ is available through the journal. The RNAseq dataset is previously published^9^ and accessible through the European Genome-phenome Archive (EGA). All accession numbers and DOIs are listed in the key resources table. The entire proteomic, transcriptomic,^9^ exercise and MRI^26^ data network is publicly available on the interactive database iNetModels 2.0^59^ (https://inetmodels.com) under “Multi-Omics Network” → “Study-specific Networks” → “Long-Term Exercise (Emanuelsson et al. 2023)”.
• All original code has been deposited at Zenodo and is publicly available as of the date of publication. DOIs are listed in the key resources table.
• Any additional information required to reanalyze the data reported in this paper is available from the lead contact upon request.

### Experimental model and study participant details

#### Study participants

In total 44 subjects (females: n = 15; males: n = 29, between 34 and 53 years old, 43 of Caucasian decent and 1 of south Asian descent) volunteered to participate in this study. Subjects went through a screening process including a health and physical activity questionnaire and a performance testing (Figure 1A), see Chapman et al. (2020) for full details. In brief, if a participant met the inclusion criteria based on the questionnaire, they were asked to perform the performance testing consisting of a maximal isokinetic knee extension test at 90°/s using the Biodex Isokinetic System (System 4 pro, Biodex medical systems) and an incremental maximal oxygen uptake test (VO_2_-peak test) on a cycle ergometer. Subjects were included into one of the following three groups if they met the following inclusion criteria: 1. endurance trained males (ME: n = 9) or females (FE: n = 9) performing vigorous endurance training on average at least three times per week over the last 15 years and displaying a VO_2_-peak within the 90^th^ percentile for their age,^78^ 2. strength trained males (MS: n = 9) or female performing resistance training on average at least three times per week over the last 15 years, a VO_2_-peak below the 90^th^ percentile for their age and a maximal torque output at least two standard deviations above the control group and had to be stronger than all endurance subjects, or 3. untrained healthy males (MC: n = 9) or females (FC: n = 8) performing any exercise on average less than 2 times per week with a VO_2_-peak below the 75^th^ percentile for their age.^78^ Unfortunately, despite our efforts we were unable to recruit strength trained females performing resistance exercise on average at least 3 times per week over the last 15 years. Trained individuals performing both endurance and resistance exercise regularly were excluded. Additional exclusion criteria were BMI >26, chronic disease, smoking, steroid or other performance enhancing drugs. Forty of the participants were included in a previously published study investigating the resting skeletal muscle transcriptome, subject characteristic, basic muscle characteristics and transcriptomic data are fully described in Chapman et al.^9^ Transcriptomic data from the remaining four subjects are published in a separate publication.^48^ Additionally, 36 individuals (ME, n = 8; FE, n = 7; MS, n = 7; MC, n = 8 and FC, n = 6) performed a whole-body MRI scan, with results previously published.^26^ All subjects went through identical screening procedures. When we discuss ‘sex differences’ in this article, we are referring to differences associated with the self-reported sex assigned at birth of our research subjects.

This study was approved by the Regional Ethical Review Board in Stockholm, Sweden (application 2016/590-31) and conformed to the Declaration of Helsinki. Prior to performing the experiment, each subject was provided a detailed description of all procedures as well as potential complications. Written and verbal consent were both attained, and the subjects were informed that they may withdraw consent at any time during the experiment.

### Method details

#### Skeletal muscle biopsy collection

Resting skeletal muscle biopsies were collected from the *M.* vastus lateralis. Participants were instructed to eat a standardized breakfast 3 h before sample collection and to refrain from any exercise the last 72 h, alcohol the last 48 h and caffeine on the morning prior to skeletal muscle biopsy collection. Following injection of local anesthesia into the skin, skeletal muscle biopsies were collected using a 5 mm Bergström biopsy needle with suction. Muscle samples were snap-frozen in liquid nitrogen-cooled isopentane and stored at −80°C until further analysis.

#### Sample preparation and mass spectrometry

Frozen skeletal muscle samples were dissolved in 500 μL Lysis buffer (4% SDS, 50 mM HEPES pH 7,6, 1 mM DTT), heated to 95°C and homogenized using a Retsch homogenizer with metallic spheres. The samples were then sonicated and centrifuged. The total protein amount was estimated (Bio-Rad DC). Samples were then prepared for mass spectrometry analysis using a modified version of the SP3 protein clean-up and a digestion protocol,^79^^,^^80^ where proteins were digested by LycC and trypsin (sequencing grade modified, Pierce). In brief, 200 μg protein from each sample was alkylated with 4 mM Chloroacetamide. Sera-Mag SP3 bead mix (20 μL) was transferred into the protein sample together with 100% Acetonitrile to a final concentration of 70%. The mix was incubated under rotation at room temperature for 18 min. The mix was placed on the magnetic rack and the supernatant was discarded, followed by two washes with 70% ethanol and one with 100% acetonitrile. The beads-protein mixture was reconstituted in 100 μL LysC buffer (0.5 M Urea, 50 mM HEPES pH: 7.6 and 1:50 enzyme (LysC) to protein ratio) and incubated overnight. Finally, trypsin was added in 1:50 enzyme to protein ratio in 100 μL 50 mM HEPES pH 7.6 and incubated overnight. The peptides were eluted from the mixture after placing the mixture on a magnetic rack, followed by peptide concentration measurement (Bio-Rad DC Assay). The samples were then pH adjusted using TEAB pH 8.5 (100 mM final conc.), 100 μg of peptides from each sample were labeled with isobaric TMT-tags (TMT10plex reagent) according to the manufacturer’s protocol (Thermo Scientific), and separated by immobilized pH gradient - isoelectric focusing (IPG-IEF) on 3–10 strips as described previously.^81^

Of note, the labeling efficiency was determined by LC-MS/MS before pooling of the samples. For the sample clean-up step, a solid phase extraction (SPE strata-X-C, Phenomenex) was performed and purified samples were dried in a SpeedVac. An aliquot of approximately 10 μg was suspended in LC mobile phase A and 1 μg was injected on the LC-MS/MS system.

Online LC-MS was performed as previously described.^81^ Using a Dionex UltiMate 3000 RSLCnano System coupled to a Q-Exactive-HF mass spectrometer (Thermo Scientific). Each of the 72 plate wells was dissolved in 20ul solvent A and 10ul were injected. Samples were trapped on a C18 guard-desalting column (Acclaim PepMap 100, 75 μm × 2 cm, nanoViper, C18, 5 μm, 100 Å), and separated on a 50 cm long C18 column (Easy spray PepMap RSLC, C18, 2 μm, 100 Å, 75 μm × 50 cm). The nano capillary solvent A was 95% water, 5%DMSO, 0.1% formic acid; and solvent B was 5% water, 5% DMSO, 95% acetonitrile, 0.1% formic acid. At a constant flow of 0.25 μL min−1, the curved gradient went from 6 to 8% B up to 40% B in each fraction in a dynamic range of gradient length, followed by a steep increase to 100% B in 5 min. FTMS master scans with 60,000 resolution (and mass range 300–1500 m/z) were followed by data-dependent MS/MS (30 000 resolution) on the top 5 ions using higher energy collision dissociation (HCD) at 30% normalized collision energy. Precursors were isolated with a 2 m/z window. Automatic gain control (AGC) targets were 1e6 for MS1 and 1e5 for MS2. Maximum injection times were 100 ms for MS1 and 100 ms for MS2. The entire duty cycle lasted ∼2.5 s. Dynamic exclusion was used with 30 s duration. Precursors with unassigned charge state or charge state 1 were excluded. An underfill ratio of 1% was used.

Orbitrap raw MS/MS files were converted to mzML format using msConvert from the ProteoWizard tool suite.^82^ Spectra were then searched using MSGF+ (v10072)^83^ and Percolator (v2.08),^84^ where search results from 8 subsequent fraction were grouped for Percolator target/decoy analysis. All searches were done against the human protein subset of ENSEMBL_101 homo sapiens protein database in the Galaxy platform.^85^ MSGF+ settings included precursor mass tolerance of 10 ppm, fully-tryptic peptides, maximum peptide length of 50 amino acids and a maximum charge of 6. Fixed modifications were TMT-10plex on lysines and peptide N-termini, and carbamidomethylation on cysteine residues, a variable modification was used for oxidation on methionine residues. Quantification of TMT-10plex reporter ions was done using OpenMS project’s IsobaricAnalyzer (v2.0).^86^ PSMs found at 1% FDR (false discovery rate) were used to infer gene identities.

Protein quantification by TMT10plex reporter ions was calculated using TMT PSM ratios to the entire sample set (all 10 TMT-channels) and normalized to the sample median. The median PSM TMT reporter ratio from peptides unique to a gene symbol was used for quantification. Protein false discovery rates were calculated using the picked-FDR method using gene symbols as protein groups and limited to 1% FDR.^87^

### Quantification and statistical analysis

#### Bioinformatics

Differential protein expression (DEP) analysis was performed using the DEqMS software package,^88^ with a false discovery rate (FDR) set to 5%. The ensembl protein id (ENSP) was used to determine the number of unique proteins. However, in some cases multiple ENSPs had the same gene name due to isoform differences. Functional analyses of the proteomic data were performed using the PIANO^31^ package in R and GSEAPY^74^ package in python 3.7 with Enrichr libraries to identify the enriched KEGG^75^ pathways and gene ontology (GO) terms. The background data used for enrichment analyses consisted of all identified proteins (n = 6713) from the current proteomics dataset. To define a process or pathway as significant, we used a cut off of FDR ≤0.05 for the distinct direction of PIANO (both up and down). Transcriptomics data were retrieved from previous publication.^9^

#### Mitochondrial proteins

To investigate known mitochondrial proteins, MitoCarta 3.0 (“Known Mitochondrial”) and the Integrated Mitochondrial Protein Index (IMPI; “IMPI Class = Verified mitochondrial” and “SVM prediction = Predicted Mitochondria High”; version IMPI-2021-Q4pre), were used.^33^^,^^34^ No tissue-specific filter was applied to this analysis because several mitochondrial proteins were still detected following the removal of proteins previously found specifically in skeletal muscle. Pathway analysis was made based on the MitoPathways3.0^33^ using the fGSEA package.^76^ To define a pathway as significant, we used a cut off of FDR ≤0.05.

#### Correlation analysis of gene-protein pairs

All detected proteins and highly expressed genes (transcripts per million [TPM] >5, n = 6979) were included for analysis.^59^ Missing values were deleted in a pairwise manner (i.e., if the gene encoding an identified protein was not detected the protein was removed from the analysis, or vice versa), which resulted in a total of 4,597 gene-protein pairs. An FDR of ≤0.05 indicated a significant correlation.

Comparisons of differentially expressed proteins and genes were performed based on gene names associated with the respective ensembl protein id (ENSP) and ensemble gene id (ENSG). The acute bout of cycling exercise consisted of a 5 min warmup at 50 W (W) followed by 30 min of cycling on a bicycle ergometer at 75% of Wmax, measured during an maximal oxygen uptake test as previously described.^9^ The endurance-trained and untrained males were included based on the same criteria as in the current study.

#### Network analysis

Network analysis was performed as previously described in iNetModels 2.0.^59^ In brief, each dataset (proteomic and transcriptomic) was pre-processed independently before network generation. Proteins were filtered out, and excluded from the network analysis, if a protein was expressed in less than 90% of the samples. Lowly expressed genes, with gene transcripts per million of <1, were excluded from the network analysis. Data from both the proteomic and transcriptomic datasets were combined into a matrix and analyzed by Spearman’s rank correlation function from SciPy package. Furthermore, we performed community detection analysis using the Leiden algorithm in the iGraph package to identify sub-network clusters for downstream analysis.

#### Cross-study comparative analysis

External studies that investigated changes in the skeletal muscle proteome in response to metabolic conditions were chosen for cross-study comparative analyses with our study. Chosen studies investigated skeletal muscle proteome in type 2 diabetes,^61^ and aging,^19^ as well as a study on high-intensity interval training (HIIT),^16^ used as a positive control. Published lists of differentially expressed proteins (DEPs) were collected from each external study and compared to the matching group of subjects from our study. If the study didn’t include a separation on the basis of sex, the external data was compared to an “All Endurance” group that included all DEPs from male and female endurance groups. Proteins were matched based on the accession number.

Cross-study comparisons were first performed through directional comparisons, analyzing the count and direction of shared DEPs between external datasets and corresponding long-term exercise data. The expression levels of shared DEPs were further analyzed to understand the general regulatory trends in response to exercise and metabolic conditions. The correlation analyses of the DEPs were done using the Spearman correlation package from SciPy on Python 3.7.

#### Statistical analysis

Sex-specific subject characteristics were calculated using one-way ANOVA and an unpaired *t* test for the male and female comparisons, respectively. Tukey’s post hoc test was used in case of statistically significant group differences from the ANOVA. The statistical analyses related to subject characteristics were performed using GraphPad Prism 8 (GraphPad, Prism, RRID:SCR_002798). Skeletal muscle proteomic and transcriptomic analyses were performed as previously described, see section “bioinformatics”.

## Supplemental information

Document S1. Figures S1‒S4

Table S1. List of all differentially regulated proteins, related to Figures 1 and 2

Table S2. List of all gene-protein pairs, related to Figure 3

Table S3. List of top 1% genes and proteins in the centrality analysis, related to Figure 4

Table S4. List of proteins in the CS activity/VO2 peak sub-network, related to Figure 4

Table S5. List of correlations and differentially expressed proteins comparing the current study with previously published datasets, related to Figure 5
