# Supplementary material for: Remodeling of the human skeletal muscle proteome found after long-term endurance training but not after strength training
Source: iScience. 2023 Dec 5;27(1):108638. doi: 10.1016/j.isci.2023.108638 (PMC10783619; doi:10.1016/j.isci.2023.108638)
Supplement: Document S1. Figures S1‒S4 [file mmc1.pdf]

iScience, Volume *27*

Supplemental information

Remodeling of the human skeletal muscle proteome found after long-term endurance training but not after strength training

Eric B. Emanuelsson, Muhammad Arif, Stefan M. Reitzner, Sean Perez, Maléne E. Lindholm, Adil Mardinoglu, Carsten Daub, Carl Johan Sundberg, and Mark A. Chapman

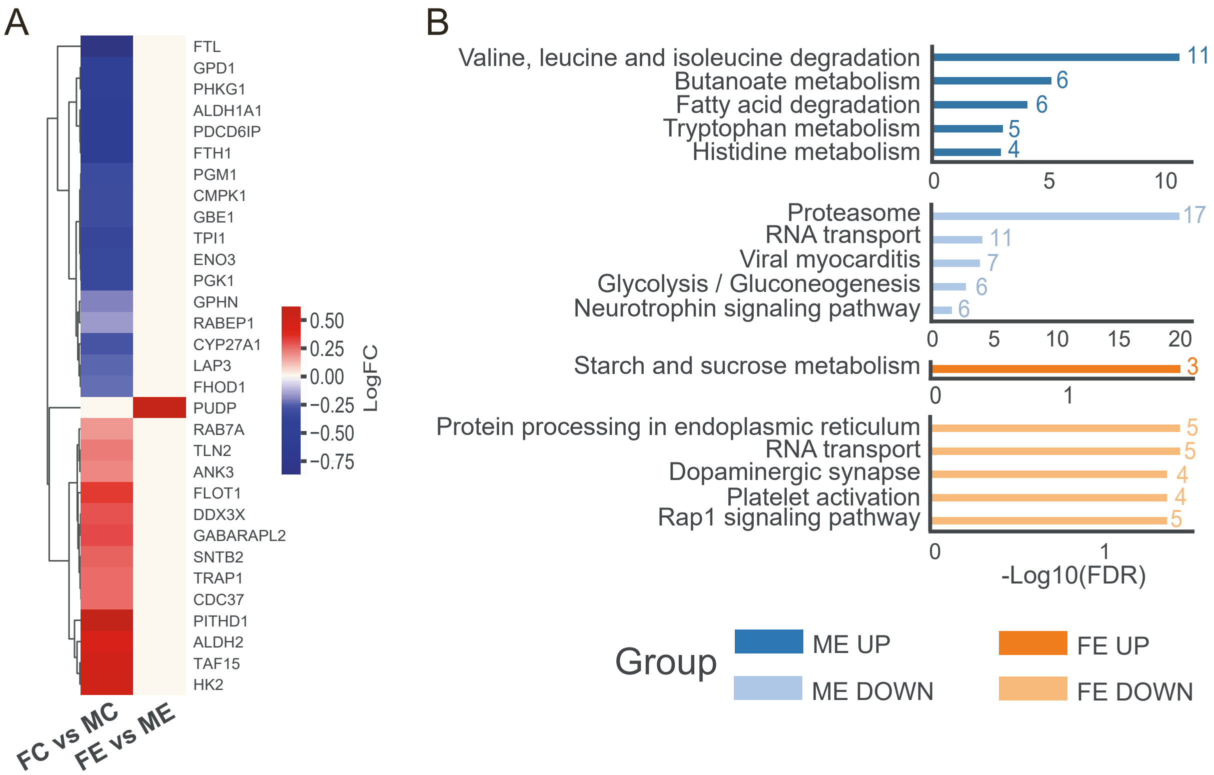

Figure S1: Sex-differences and sex-specific adaptations to long-term endurance training. A) Heatmap of all differentially expressed proteins between FC vs MC and FE vs ME. B) Sex-specific overrepresentation analysis of the proteins significantly upregulated in ME vs MC (dark blue panel) or in FE vs FC (dark orange panel) and downregulated in ME vs MC (light blue color) or in FE vs FC (light orange color). Red boxes in all heatmaps indicate a significant upregulation, blue indicate a significant downregulation. ME = male endurance, MC = male control, FE = female endurance, FC = female control, LogFC = Log fold change. Related to Figure 1 & 2.

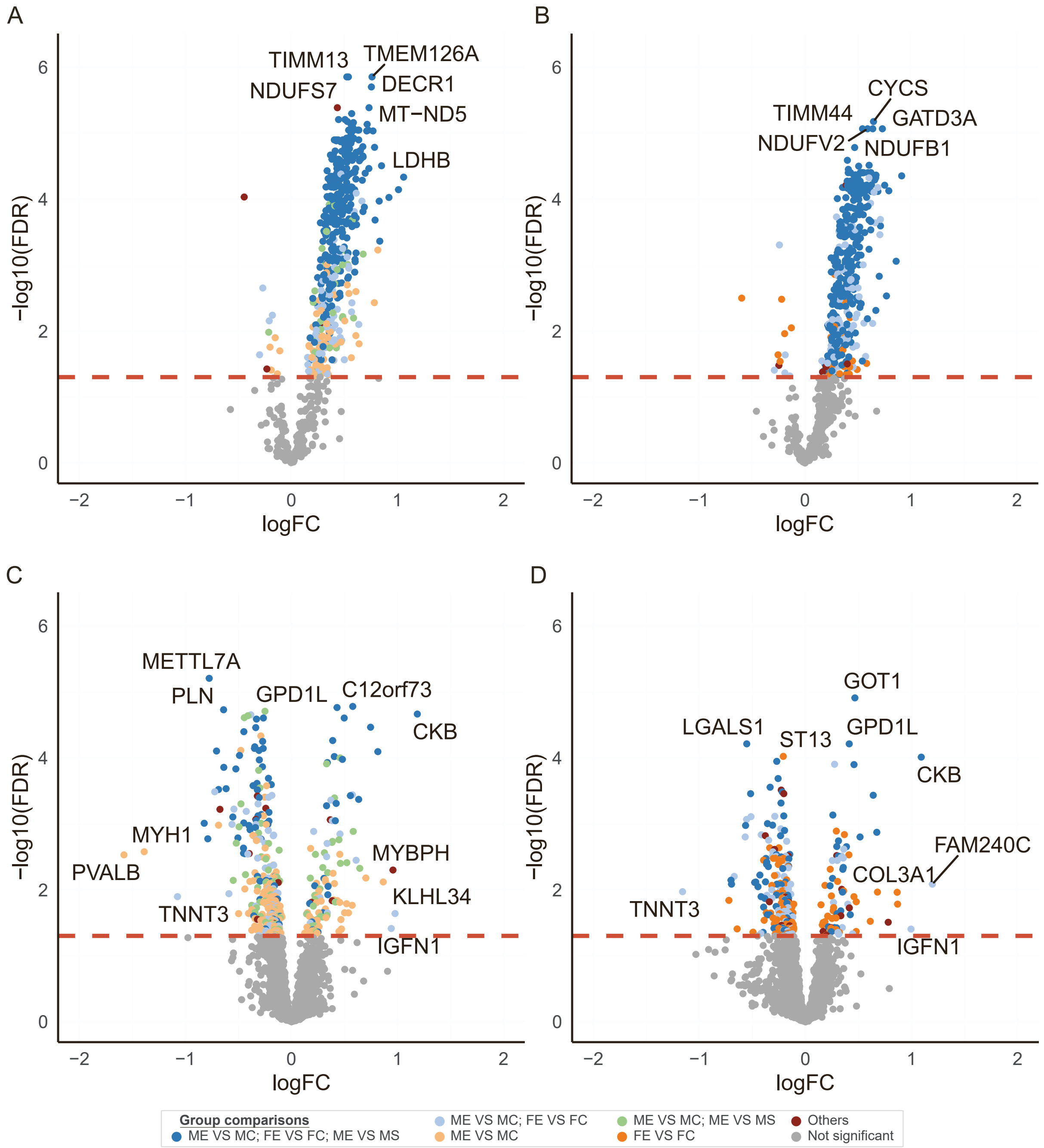

Figure S2: Mitochondrial and non-mitochondrial protein abundance. A & B) Volcano plots of all mitochondrial DEPs. C & D) Volcano plots following removal of all known mitochondrial proteins between ME vs MC and FE vs FC, respectively. The color of each dot indicates if the protein is unique to one group comparison or shared between more group comparisons. Related to Figure 2.

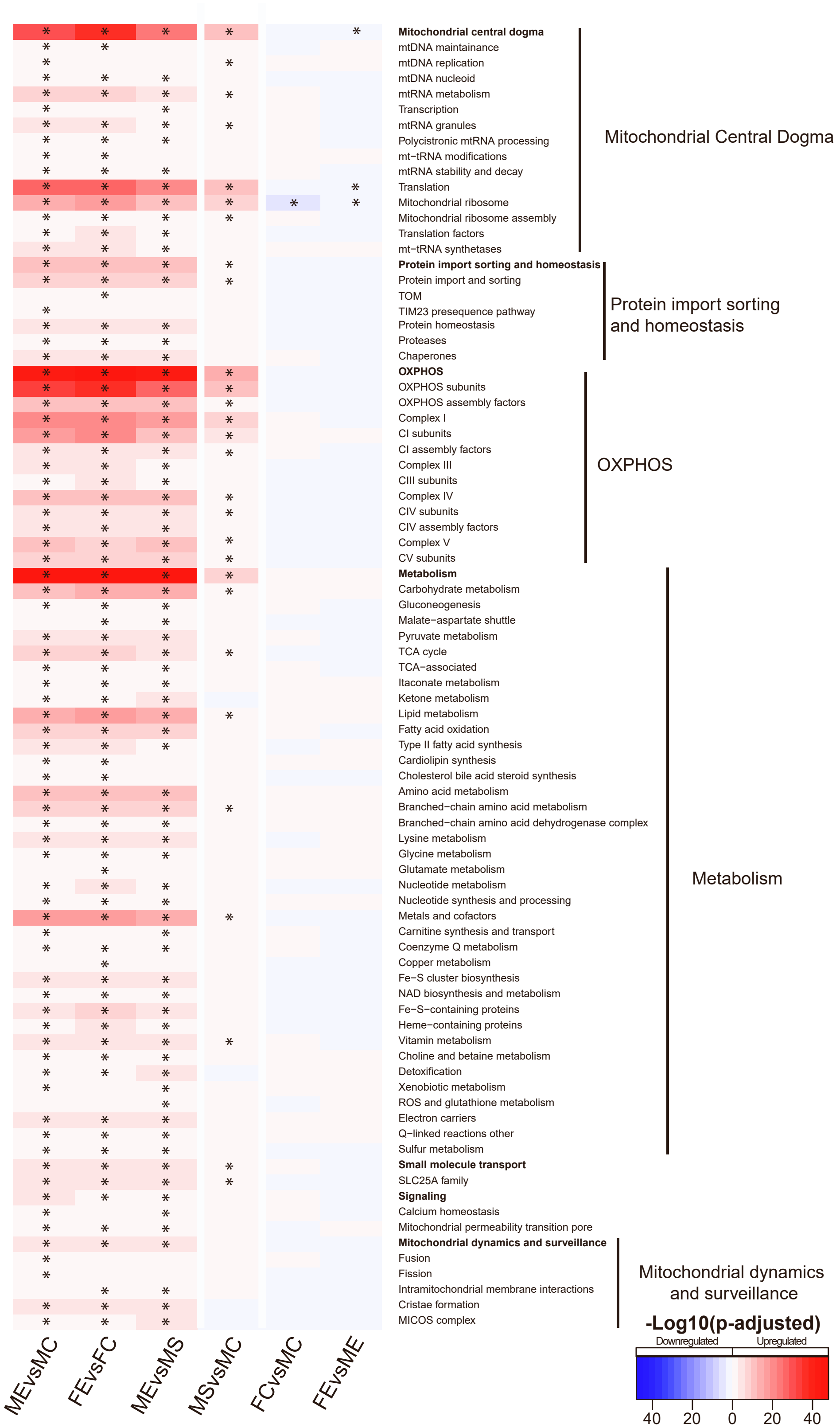

Figure S3: MitoPathways. Gene-set enrichment analysis of MitoPathways. * = statistical significant difference, FDR < 0.05. ME = male endurance, MC = male control, FE = female endurance, FC = female control and MS = male strength. Related to Figure 2.

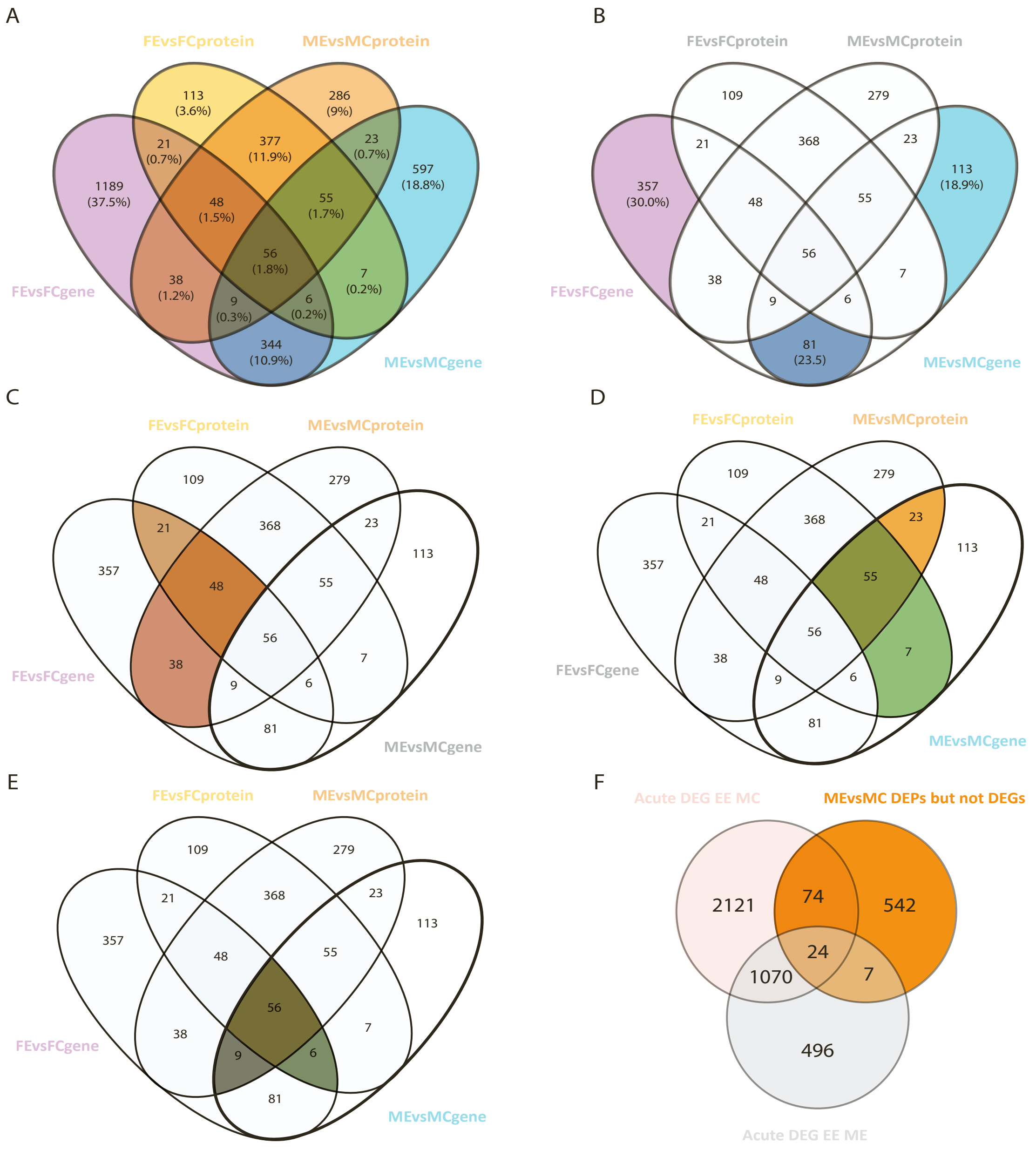

Figure S4: Overlap of differentially expressed proteins (DEP) and genes (DEG). A) displays all DEGs and DEPs between ME vs MC and FE vs FC. B) As the corresponding proteins were not identified in at least 50% of the samples of 66% of all DEGs, those DEGs where removed. C & D) Highlights the DEGs with a corresponding DEP in FE vs FC and ME vs MC, respectively. E) Highlights the shared DEGs in both FE vs FC and ME vs MC with a corresponding DEP. F) To obtain a better understanding of potential temporal differences in gene expression and protein abundance, we used preliminary data from our lab investigating gene expression following acute endurance exercise (EE) in highly endurance trained (ME) and untrained males (MC). Of the 663 DEPs identified without a corresponding DEG, a total of 105 (15.8%) additional DEP between ME vs MC were also regulated at the gene expression level within the first 3h following an acute bout of endurance exercise. Related to Figure 3.
